# Raman and FT-IR Spectroscopy investigation the cellulose structural differences from bacteria *Gluconacetobacter sucrofermentans* during the different regimes of cultivation on a molasses media

**DOI:** 10.1186/s13568-020-01020-8

**Published:** 2020-05-03

**Authors:** Nelli Atykyan, Victor Revin, Vitalina Shutova

**Affiliations:** National Research Mordovia State University, Saransk, Russia

**Keywords:** Bacterial cellulose, *Gluconacetobacter sucrofermentans*, FT-IR spectroscopy

## Abstract

Raman and Fourier Transform Infrared (FT-IR) spectroscopy was used for investigation of structural differences of bacterial celluloses (BC), obtained by cultivation native and immobilized cells of *Gluconacetobacter sucrofermentans* during static and dynamic regimes of cultivation on a molasses media. It was found that the Raman and FT-IR spectra could characterized the groups of the cellulose molecules. The culturing bacterial cellulose in the presence of results in an increase of crystalline and it increased during cultivated on a molasses media with the addition of 1.5% ethanol—75.62%. The degree of BC crystallinity increased during dynamic regime of cultivation is higher than under static regime one. The maximal BC content was observed when 0.5% ascorbic acid was added to the cultivation medium with molasses and native cells. It was found, the degree of BC crystallinity during static regime cultivation on a molasses medium with ethanol, increased significantly to 73.5%, and during dynamic regime—75.6%. So, in this study, the changes of the bacterial cellulose conformation of were revealed during bacterial cultivation in a medium containing molasses in various cultivation modes.

## Introduction

Bacterial cellulose (BC) is carbohydrates produced by *Komagataeibacter, Gluconacetobacter, Enterobacter, Pseudomonas, Achromobacter, Alcaligenes, Aerobacter, Azotobacter, Agrobacterium, Burkholderia, Dickeya, Escherichia, Rhizobium, Salmonella,* and *Sarcina* with unique biotechnological properties (the finest porosity and mechanical strength) and wide range of applications (promising material for medicine, industry and technology) (Klemm et al. 2001; Zhu et al. 2001; Maneerung et al. 2008; Cauerhff and Castro 2013; Lee et al. 2014; Augimeri et al. 2015; Cacicedo et al. 2015; Rastogi et al. 2018). Important, the plants cellulose formed complex with other compounds such as hemicelluloses, lignin etc. but BC is pure cellulose and does not contain these components (Fu et al. 2013; Augimeri et al. 2015). BC is biodegradable and non-toxic, has a high crystallinity, but is not completely crystalline, and a high degree of polymerization (Schenzel and Fischer 2004; Hsieh et al. 2008; Kaewnopparat et al. 2008; Szymańska-Chargot et al. 2011). The BC synthesis depends on by number of experimental factors: (1) the culture medium composition, namely: sources of carbon, nitrogen, micro- and macro elements; (2) the cultivation conditions (temperature, pH, dissolved oxygen concentration, cultivation method, etc.) (Matsuoka et al. 1996; Ramana et al. 2000; Thompson and Hamilton 2001; Chawla et al. 2009; Pa’e et al. 2011); (3) not only glucose and sucrose are used for BC synthesis, but fructose, maltose, xylose, starch, glycerin, ethanol and other sources of carbon can be used (Hestrin and Schramm 1954; Park et al. 2003; Hungund et al. 2013). Concerning, the carbon sources are often the most expensive components of the culture medium, so modern biotechnology offers to use sugar-containing waste from food industries, such as molasses, DDGS, whey (Keshk and Sameshima 2006; Kongruang 2008; Coban and Biyik 2011; Zeng et al. 2011; Guo et al. 2013; Rani and Appaiah 2013; Wu and Liu 2013; Jozala et al. 2014; Lin et al. 2014; Kiziltas et al. 2015). At the same time, a change in the composition of the medium and the condition of cultivation leads to an increase/decrease yield of the BC, but to modification of its structure. The BC properties depend on the degree of cellulose crystallinity. The crystalline domains in BC are of very limited size and are mixed with noncrystalline regions. The latter is important because the plant celluloses and BC have the same molecular structure, but differ in crystalline structures connected with its crystalline structure polymorphs (cellulose I–IV): in nature (cellulose I); cellulose II is synthesized by bacteria or algae. In addition, cellulose II and cellulose III and IV can be obtained from cellulose I by chemical treatment (Brett and Waldron 1990; Szymańska-Chargot et al. 2011). The cellulose crystalline domains are divided by the non-crystalline cellulose regions and the ratio of crystalline and non-crystalline regions determine some properties of cellulosic fibrils, for example tensile strength (Szymańska-Chargot et al. 2011). The ratio of crystalline regions to total fibrils of cellulose (the crystallinity index) can be investigated by a variety of methods like X-ray diffraction, solid-state 13C-NMR or wide-angle X-ray scattering experiments (Zugenmeier 2008). This method can be supplemented by infrared and Raman spectroscopy, which are the simplest and the least time consuming methods of BC crystallinity index determination (Schenzel et al. 2005). An obvious disadvantage of this method is that it can give only relative crystallinity index values because the spectrum consists of contributions from both amorphous and crystalline regions.

The purpose of this work was a comparative analysis by the method of FTIR and Raman spectroscopy of BC structure obtained by cultivation native and immobilized cells of *Gluconacetobacter sucrofermentans* during static and dynamic regimes of cultivation on a molasses media.

## Materials and methods

### Microorganisms and culture conditions

The studies object was the bacteria cellulose from the *Gluconacetobacter sucrofermentans* VKPM B-11267, selected at the Department of Biotechnology, Bioengineering and Biochemistry National Research Mordovia State University and was deposited in Russian National Collection of Industrial Microorganisms. Bacterial culture was maintained on medium of Hestrin-Shramm (Hestrin and Schramm 1954) at the 4 °C after growing them for 3 days at 28 °C. For the cultivation of producers used nutrient medium of the following composition (g/l):

1. medium of Hestrin-Shramm
2. medium with molasses (24 g/l in terms of sucrose)
3. medium with molasses (36 g/l in terms of sucrose)
4. medium with molasses (48 g/l in terms of sucrose)
5. medium with molasses (36 g/l in terms of sucrose) with addition 1.5% (v/v) ethanol
6. medium with molasses (36 g/l in terms of sucrose) with addition 1.5% (v/v) glycerol
7. medium with molasses (36 g/l in terms of sucrose) with addition 0.5% (w/v) ascorbic acid
8. medium with molasses (36 g/l in terms of sucrose) with addition 0.5% (w/v) lignosulfonate.

The initial culture for the preparation of the inoculum was a culture on a mown agar medium. It was washed and suspension was used for the inoculum preparation. A suspension of microorganisms (10 ml) were seeded in the flasks with 100 ml of medium. The inoculum was grown on an orbital shaker—20/60 incubator shaker (Biosan, Latvia) at 250 rpm and a temperature of 28 °C for one day.

The dynamic regime of bacteria cultivation was carried out in bioreactors BIOSTAT A plus (Sartorius, Germany) with mechanical (200 rpm) and air (1 l per 1 l medium per minute) mixing. Experimental flasks were seeded with 10% inoculum and placed on an ES-20/60 shaker incubator (Biosan, Latvia) at 200 rpm for 3 days. Bioreactors were also inoculated with 10% inoculum and cultured for 3–5 days. The static regime of bacteria cultivation was carried out in flat containers: a suspension of microorganisms (50 ml) was seeded with 500 ml of medium and then left in static conditions.

To remove cells and medium components BC was treated with a 0.1 M HCl at 85 °C for 30 min and 3 times washed with distilled water. After BC was treated with 1 N NaOH at 80 °C for 30 min and also 3 times washed with distilled water. The BC was boiled in water for 10 min and the amount of bacterial cellulose was determined by the gravimetric method.

### FTIR spectroscopy of bacterial cellulose samples

The BC FT-IR spectra were collected on a Fourier spectrometer model IRPrestige-21 (Shimadzu, Japan). The purified BC was prepared to additional drying to constant weight at 60 °C. The samples (2–4 mg) were fixed (in an agate mortar with 0.12 g of KBr) and compressed (laboratory hydraulic press PGR-10 at a pressure of 210 bar for 1 min). The spectra were collected over the range 4000–650 cm^−1^. For each material, five samples under the same conditions were examined. For each sample, 200 scans were averaged with a spectral resolution of 4 cm^−1^. Then for a given material, a final average spectrum was calculated. These spectra were normalized to 1.0 at 1136 cm^−1^ (COH stretching vibration). Baseline corrections were obtained on Omnic Software (Thermo Scientific).

It is known that two IR peaks around 750 and 710 cm^−1^ are characteristic for triclinic (Iα) and monoclinic unit (Iβ) allomorphs, respectively. The relative proportion of cellulose Iβ to Iα allomorph could be calculated by integrating the absorption bands near 710 and 750 cm^−1^ and the percentage of Iβ could be obtained.

### Raman spectroscopy of BC samples

Before Raman spectrum registration the BC was dried to constant mass The bacterial cellulose films was applied on a microscope glass. Raman spectra were collected on a Raman-dispersive spectrometer inVia by Renishaw (UK), based on a LeicaDM 2500 microscope with laser 532 nm and maximum power 10 mW. The spectra were recorded over the range 1700–850 cm^−1^ using an operating spectral resolution of 2.0 cm^−1^ of Raman shift. Spectra were taken with 5 s exposure and 100 mW laser power output. These spectra were normalized to 1.0 at 2900 cm^−1^. It was shown that the intensity of RS peaks at 1462 and 1481 cm^−1^, which correspond to CH2 bending, relates to crystalline/amorphous proportions in cellulosic molecules. The higher the peak at 1481 cm^−1^, the higher the cellulose crystallinity degree is too. In the case when amorphous cellulose predominates over crystalline one, there is only evidence of a broad peak at 1462 cm^−1^. On this basis one can estimate the crystalline index by counting a relative percentage amount of crystalline fraction in a cellulosic sample.

### X-ray diffraction (XRD)

X-ray diffraction (XRD) was performed using “Empyrean PANalytical” X-ray diffractrometer in the filtered radiation of a copper anode (λ = 0.15418 nm, 40 kV b 30 mA) in the range of angles 2θ from 10 to 60°. The measurements were carried out using a two-coordinate detector Pixcel 3d that operated in the linear scanning mode (255 pixels per strip) with a resolution of 0.013 degrees/strip. The sample of bacterial cellulose was a gel film of bacterial cellulose dried at 60 °C. To exclude additional scattering from the substrate, a standard holder for strong films was used. Thus, the contribution to the total scattering was determined only by scattering by air. Cellulose samples have a layered fiber structure in which the fibers lie mainly in the plane of the film. In this regard, the measurements were carried out in two setups: “reflection” and “transparency”.

### Scanning electron microscopy

The BC was analyzed by scanning electron microscope TM 3000, manufactured by HITACHI (Japan) with the microanalysis system SwiftED3000, manufactured by Oxford Instruments (UK). Microscope resolution was of the order of 50 µm (a magnification of 1600×), depth resolution—0.5 mm. Measurements of the BC were taken in a high vacuum of the order of 10^−2^–10^−3^ Pa, at an accelerating voltage of 15 kV. To pretreat the sample dry method of preparation was used.

### *Gluconacetobacter sucrofermentans* cells immobilization

The cell biomass obtained by aseptic centrifuging at 5000 rpm during 10 min was mixed at room temperature in an 8% solution of polyvinyl alcohol prepared. The suspension was distributed, frozen at − 20 °C and kept in the frozen state for 17 h. The pellets were thawed at 8 °C for 3 h.

### Statistical processing of results

Statistical treatments of experimental data carried out by the method of variation statistics on a personal computer using Microsoft Excel.

## Results

**Fig. 1:**
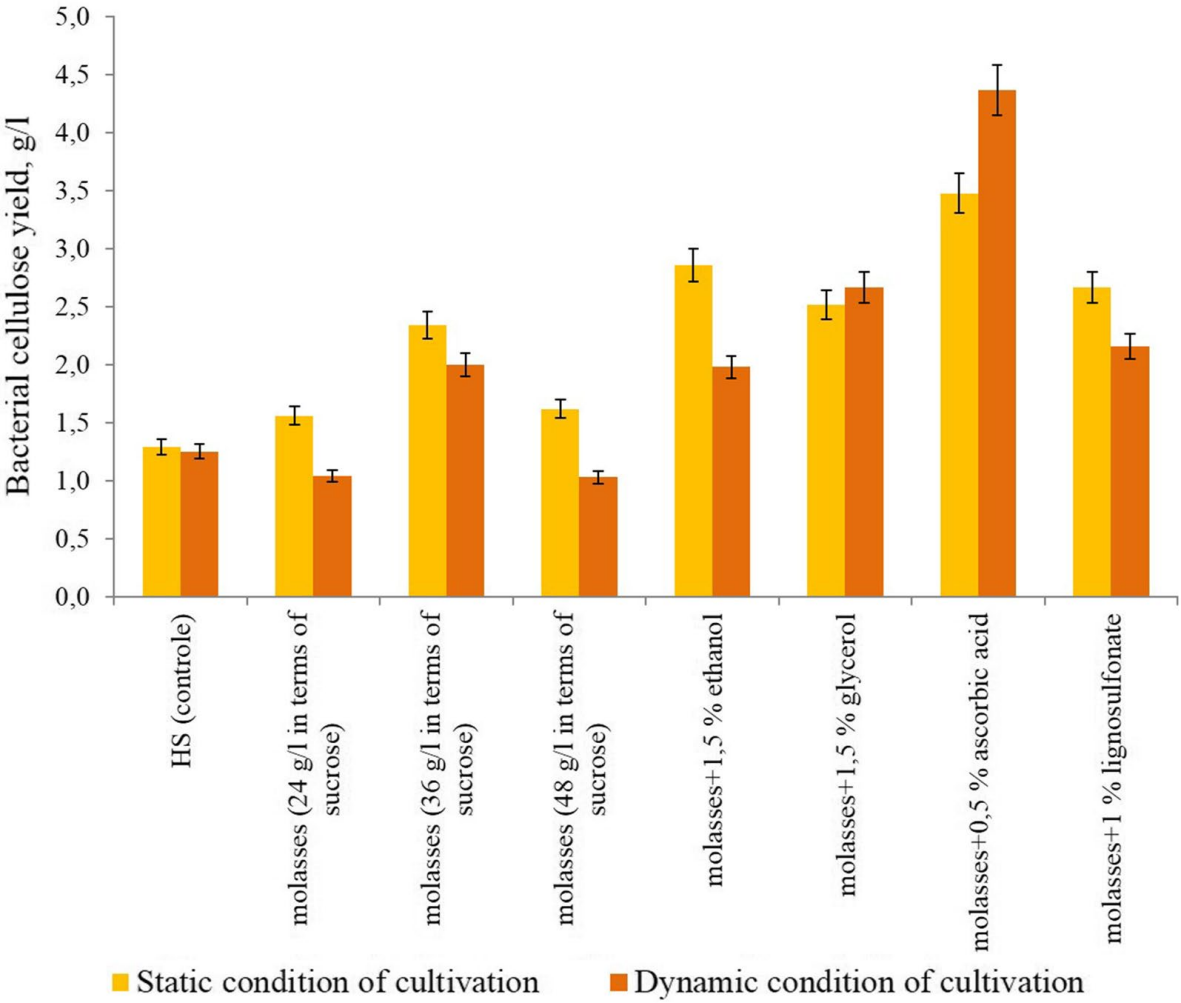
Yield of bacterial cellulose under static and dynamic condition of cultivation on various medium

During the bacteria cultivation on HS medium the content of BC was 1.25 g/l during static and dynamic regimes (Fig. 1). Then the bacteria cultivation was carried out on a medium with molasses. It was found that during cultivation on molasses (50 g/l) medium the BC contents was increased in static regime than in dynamic ones (1.56 g/l). When the molasses concentration changes to 75 g/l the BC contents was 2.34 g/l (in static regime) and 2 g/l (in dynamic regime) and increasing of the molasses concentration did not have a positive effect on BC contents. In next experiments we used 1.5% ethanol with 75 g/l molasses. In this case, the BC output during static regime was 2.86 g/l, and in dynamic—1.98 g/l (Fig. 1). In next stages of investigation as an additive in molasses medium was used ascorbic acid (0.5% w/v). The cellulose production during static regime was 2.48 ± 0.12 g/l, and in during dynamic one—3.73 ± 0.18 g/l (Fig. 1)

**Fig. 2:**
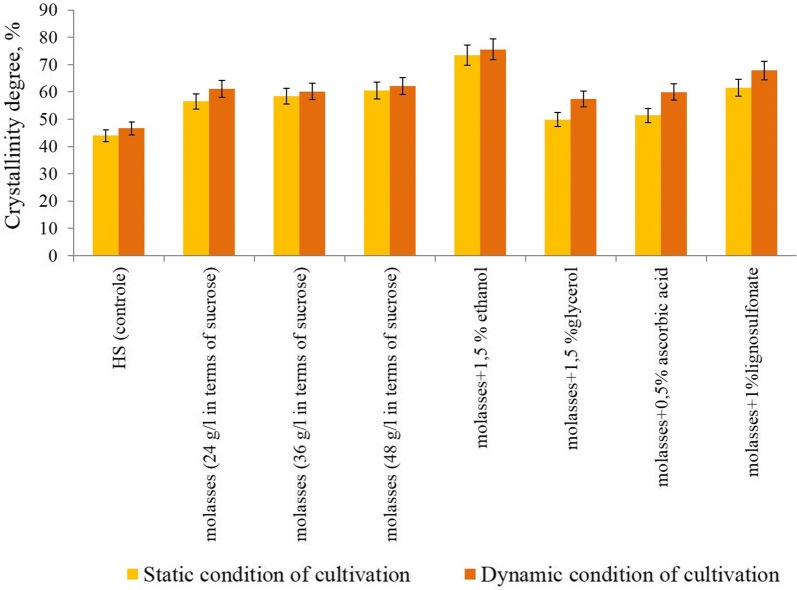
Crystallinity degree of bacterial cellulose obtained in static and dynamic condition of cultivation on various medium

According this investigation general idea in next experiments was to study the changes of the degree of BC crystallinity using IR and RS. It has been shown that no changes in the of BC degree of crystallinity in HS medium at different conditions (Fig. 2). It was found, the degree of BC crystallinity during static regime cultivation on a molasses medium with ethanol, increased significantly to 73.5%, and during dynamic regime—75.62% (Fig. 2). During static regime cultivation the bacteria on molasses medium with the ascorbic acid, the BC degree of crystallinity was 51.40% (lower than in the control), and during dynamic regime—59.92% (approximately at the same level as in the control).

**Fig. 3:**
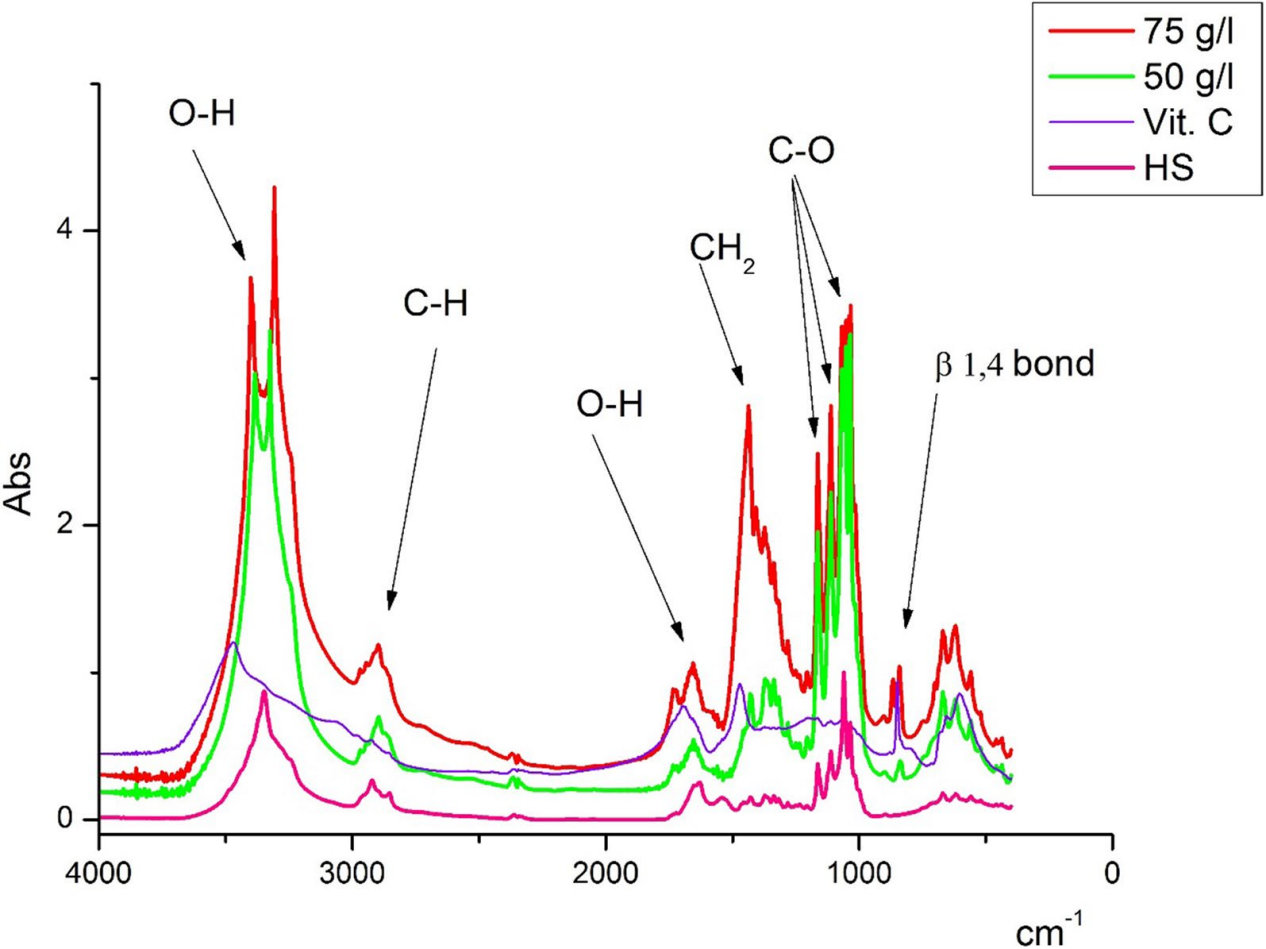
FTIR spectra of bacterial cellulose obtained in static and dynamic condition of cultivation on various medium

In next experiment the BC FTIR spectrum at the different of cultivation medium composition during static regime was studied. The BC FTIR spectra in these experiments does not changes. Figure 3 shows the FTIR spectra of BC obtained by bacteria cultivation at static and dynamic regime on a molasses and HS medium with the addition of ethanol and ascorbic acid. As we can see the FTIR spectra are identical—there is only a slight shift of the peak correlated with the valence vibrations of the OH groups in BC obtained on molasses medium with addition of ascorbic acid.

**Fig. 4:**
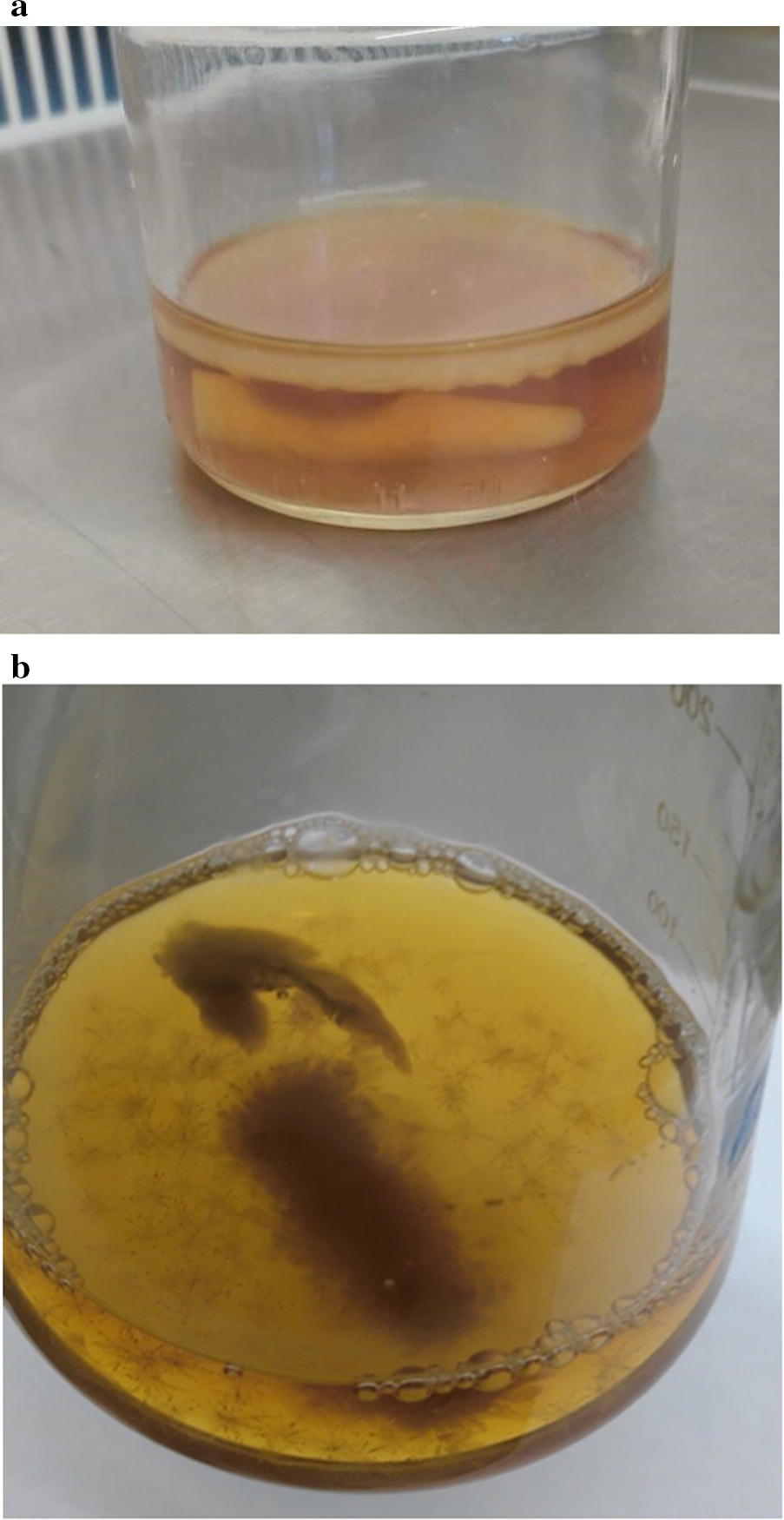
Immobilized cells producing bacterial cellulose (HS medium): **a** static conditions of cultivation, **b** dynamic conditions of cultivation

**Fig. 5:**
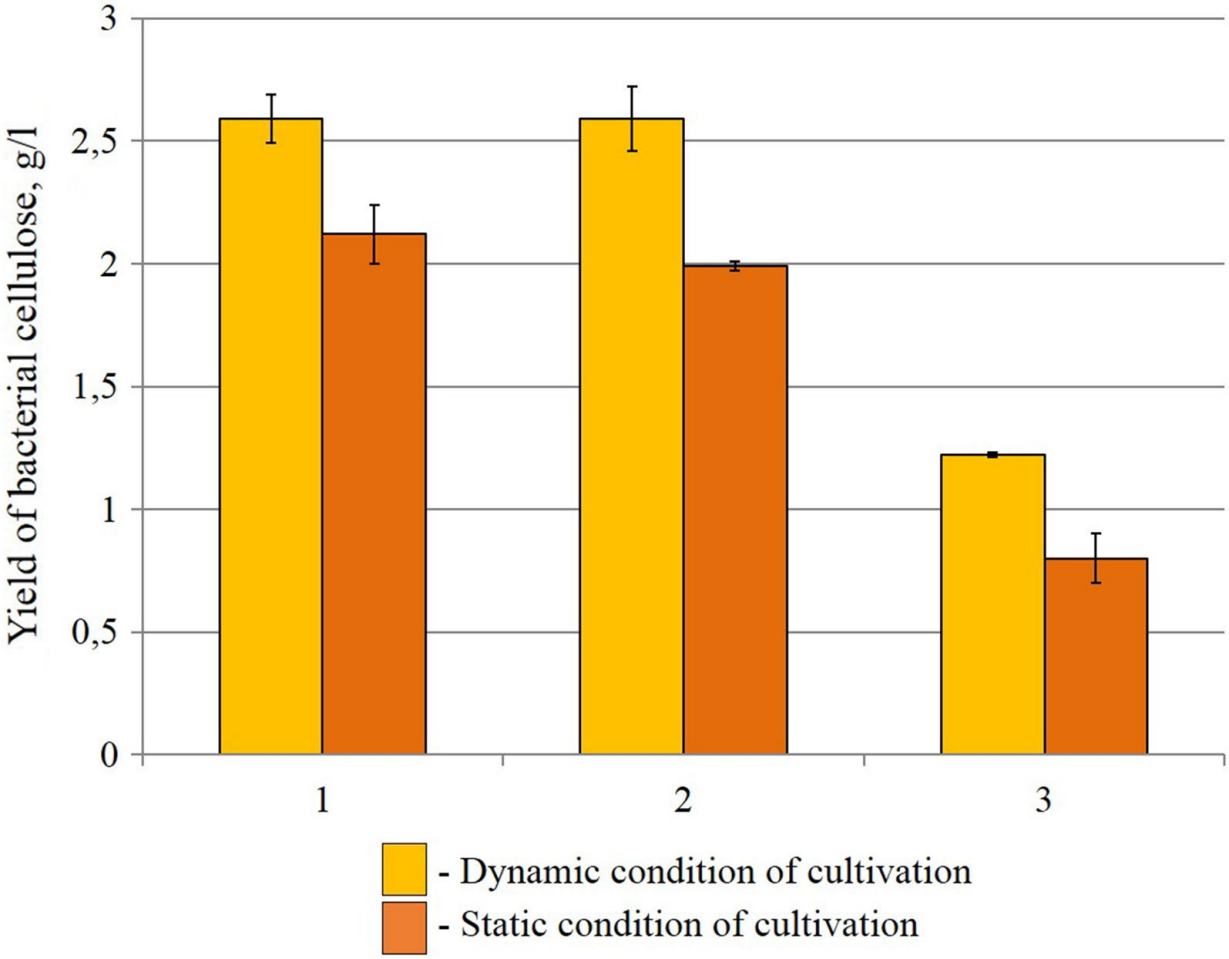
The yield of bacterial cellulose obtained in static and dynamic conditions of semi-continuous cultivation using immobilized cells

**Fig. 6:**
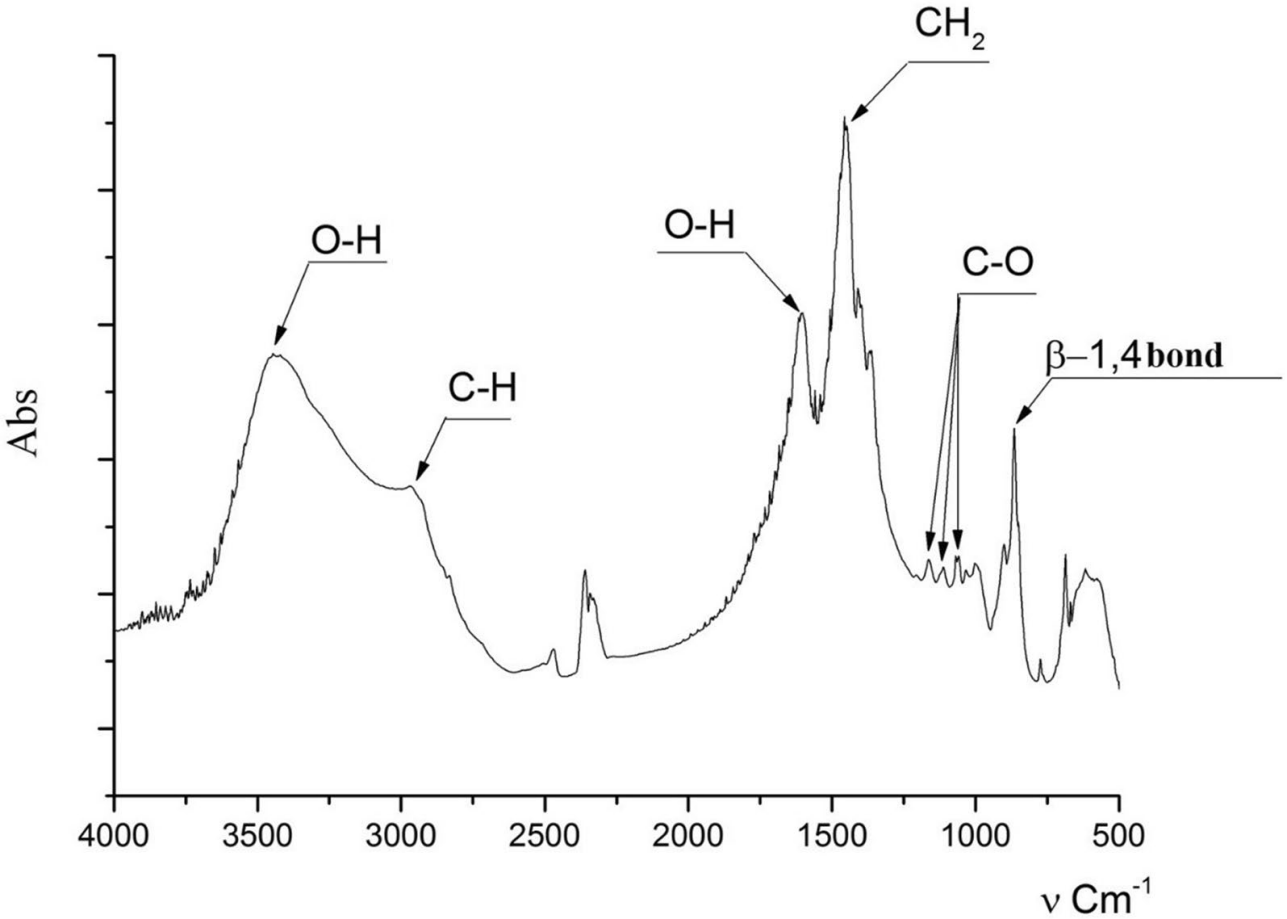
FTIR spectrum of bacterial cellulose obtained under dynamic conditions on a molasses medium using producer immobilized cells

**Fig. 7:**
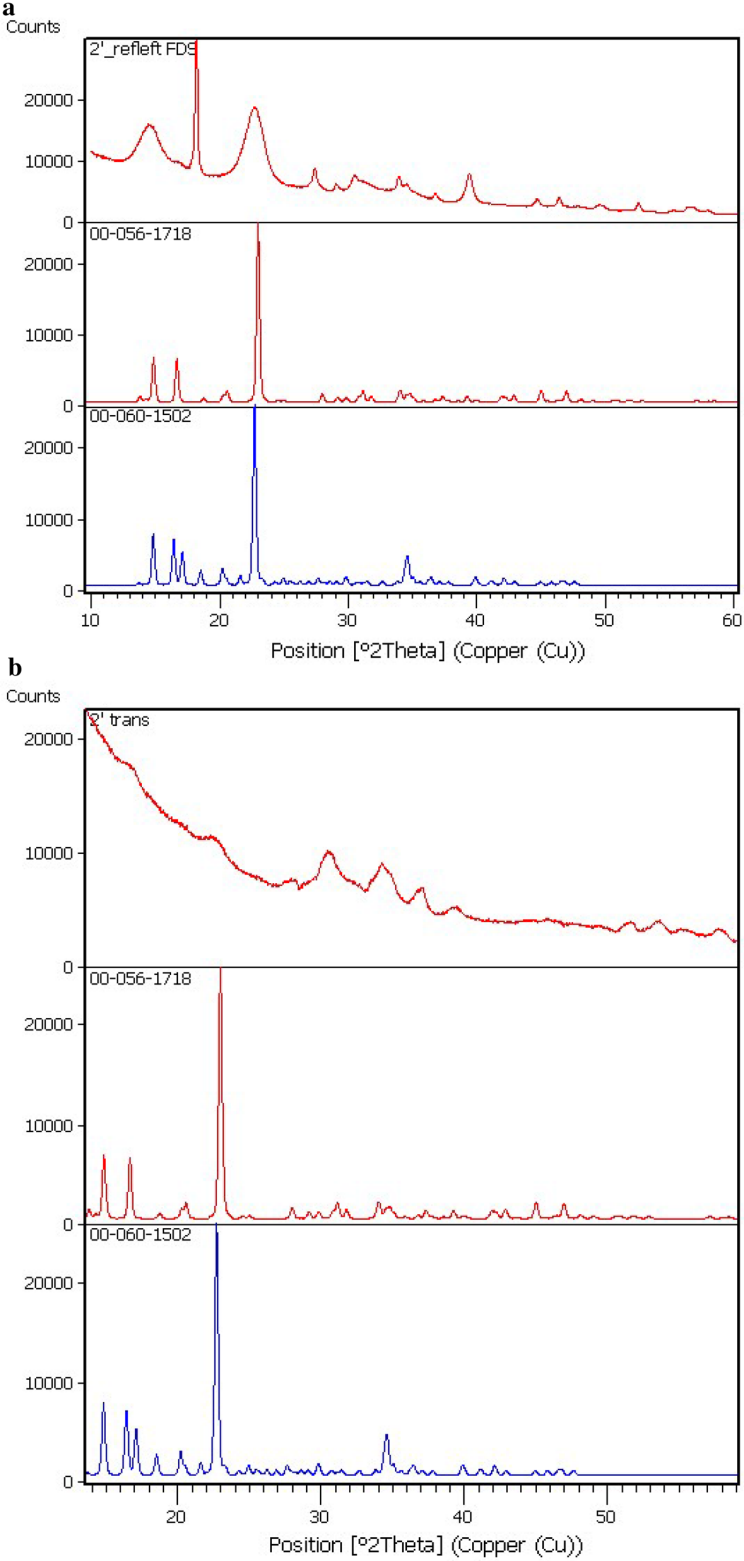
X-ray diffractogram obtained from the BC sample (measurements were carried out in two setups: “reflection” (**a**) and “transparency” (**b**))

**Fig. 8:**
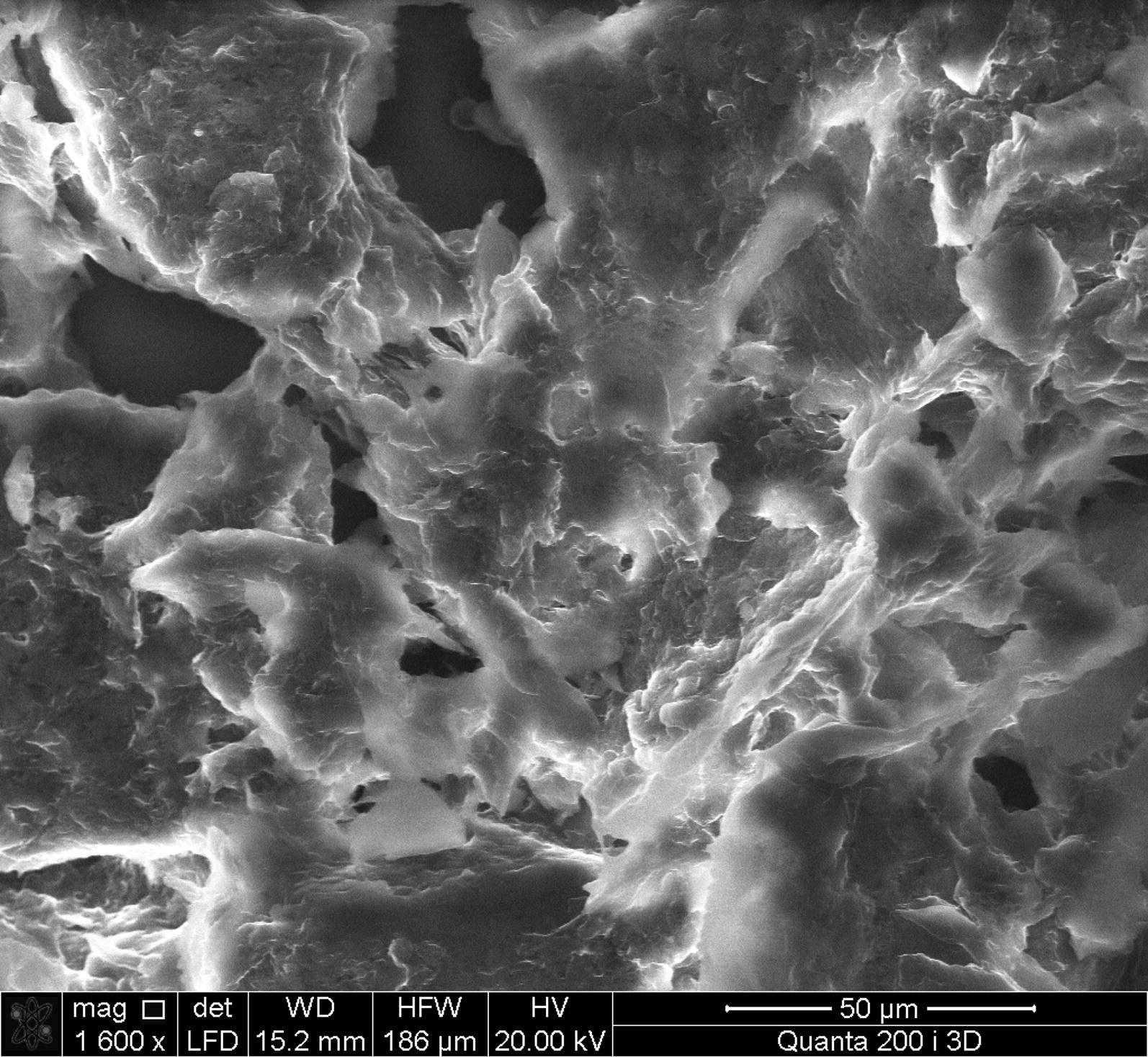
SEM images of dried flakes of bacterial cellulose synthesized by immobilized culture

In next experiments was used immobilized cells of bacteria. Our investigation was demonstrated that during the static cultivation regime using immobilized cells in a cryogel the BC is formed film on the surface of the medium (Fig. 4a), and during dynamic regime—in form of “stars” in small cellulose flakes (Fig. 4b). Using the immobilized cells, semi-continuous cultivation of the BC was carried out at static and dynamic regimes. It was found that at the first period of cultivation during static regime, the maximal BC contents was 2.12 ± 0.12 g/l, but during the second and third period, it decreased to 1.99 ± 0.02 g/l, and 0.80 ± 0.10 g/l, respectively (Fig. 5). During the dynamic regime of the cultivation at the first and second stage, the BC output does not change (2.59 ± 0.13 g/l), but, there BC contents significant decreased in the third period (to 1.22 ± 0.01 g/l). Using the FTIR the molecular structure of BC obtained with help of immobilized cells during dynamic and static culture conditions regime were the same (Fig. 6). In next experiment we use RS for studies changes of the degrees of crystallinity BC production with the help of immobilized *Gluconacetobacter sucrofermentans* cells; the degree of crystallinity of the BC was approximately the same 66% (Dynamic regime) and 52.26% (Static regime). A typical X- ray diffractogram obtained from the BC sample demonstrated two characteristic clearly resolved peaks (Fig. 7). SEM images of dried samples of flakes of bacterial cellulose was received (Fig. 8). It can be seen from the image that, as in the case of synthesis by native bacteria, during synthesis by an immobilized culture, BC is formed in the form of flakes consisting of interweaving of thinner fibers.

So, in the course of this study, the changes of the bacterial cellulose conformation of were revealed during bacterial cultivation in a medium containing molasses in various cultivation modes.

## Discussion

During the bacteria cultivation on HS medium the yields of BC was small as during static also in dynamic regime which may be due to the accumulation of gluconic acid in the process of utilization of glucose by G. sucrofermentans (Masaoka et al. 1993). Moreover, HS medium includes expensive components as glucose, yeast extract, peptone, etc. In this reason the next experiments the bacteria cultivation was carried out on a cheap medium with molasses, which as was shown has a positive effect on the synthesis of BC due to the polyphenols and sucrose (Coban and Biyik 2011; Zeng et al. 2011). Molasses is traditionally used for cultivation of bacteria producing various polysaccharides—alginate (Revin et al. 2018), levan (Revin et al. 2016), dextran (Revin and Shutova 2015) and others. Also it is known that during bacteria cultivation in media with molasses, the changes of reducing sugars content leads to a decrease the gluconic acid production (Zeng et al. 2011). In our experiments changing HS medium on molasses (75 g/l) increased the yield of BC more than 1.5 time and 2 time in molasses medium with ethanol. Probably adding ethanol to the medium increased cellulose production and decreased the numbers of mutants non-cellulose producing cells (Park et al. 2003; Son et al. 2003). Also was shown that BC synthesis increases with the addition of antioxidants, for example, vitamin C (Keshk 2014a, b). The yield of bacterial cellulose in molasses medium with adding 0.5% ascorbic acid increased 3 time approximately at the yields in the control HS medium.

The BC RS peaks at 1462 cm^−1^ and 1481 cm^−1^ correspond to CH_2_ bending, relates to crystalline/amorphous proportions in some cellulosic molecules (Schenzel et al. 2005). It was observed that a higher peak at 1481 cm^−1^ corresponds with a higher degree of crystallinity. It has been shown that no changes in the of BC degree of crystallinity in HS medium at different conditions but on a molasses medium with ethanol, crystallinity was increased significantly to 75.62% (Dinamic regime).

The FTIR spectrum peak in the region of 3347 cm^−1^ characterizes the stretching vibrations of OH groups, the peak in the region of 2897 cm^−1^ indicates the stretching vibrations of the C–H groups and 1662 cm^−1^ is the region of deformational vibrations of OH-groups of bound water. The BC FTIR spectrum peaks in the range of 1500–1200 cm^−1^ are sensitive to chemical and molecule structural transformations. The IR bands in the region of 1000–1200 cm^−1^ are connected with stretching C–O–C and C–O vibrations. The IR peak in the region of 847 cm^−1^ characterizes the β-1,4 bonds vibrations. The BC FTIR spectra in different experiments practically do not change.

As known, immobilization is a technique used to trap the cells into a matrix. The aim of this stage was using immobilized cells for the fermentation of BC repeatedly. There is a data about using of immobilized cells of *Acetobacter xylinum* for the production of nata de coco is one alternative to the product resulted in a cell-free nata. From the results obtained that immobilized cell still produced nata up to two replications fermentation (Nugroho and Pradipta 2015). Our investigation was demonstrated that during the static cultivation regime using immobilized cells in a cryogel the BC is formed film on the surface of the medium, and during dynamic regime—in form of “stars” in small cellulose flakes. Using the FTIR the molecular structure of BC obtained with help of immobilized cells during dynamic and static culture conditions regime were the same. In next experiment we use RS for studies changes of the degrees of crystallinity BC production with the help of immobilized *Gluconacetobacter sucrofermentans* cells; the degree of crystallinity of the BC was lower than in BC from native cells of producers. X-ray diffractogram investigations indicated that majority of the cellulose synthesized as by native also by immobilized culture was type-1β cellulose what typical for Gluconacetobacter sp.

So, in the course of this study, the changes of the bacterial cellulose yields of were revealed during bacterial cultivation in a medium containing molasses with ascorbic acid and when we use immobilization of producer.

## Data Availability

All data are fully available without restriction.
